# Measurement Instruments Used to Assess Caregiver Burden in Informal Caregivers of Adult Cancer Patients: A Systematic Review

**DOI:** 10.3390/nursrep16050172

**Published:** 2026-05-19

**Authors:** Valentina Cerrone, Rocco Capasso, Marco Cascella, Ivan Rubbi, Anna Di Gisi, Pierpaolo Di Santo, Vincenzo Andretta

**Affiliations:** 1 University Oncology Clinic Unit, University Hospital “San Giovanni di Dio e Ruggi D’Aragona”, 84131 Salerno, Italy; valentina.cerrone@sangiovannieruggi.it; 2 Oncology Unit, Istituto Nazionale Tumori IRCCS “Fondazione Pascale”, 80131 Napoli, Italy; r.capasso@istitutotumori.na.it; 3 Department of Medicine, Surgery and Dentistry “Scuola Medica Salernitana”, University of Salerno, 84081 Baronissi, Italy; mcascella@unisa.it; 4 School of Nursing, University of Bologna, 48018 Faenza, Italy; ivan.rubbi2@unibo.it; 5 UOC SPS Direzione Sanitaria, University Hospital “San Giovanni di Dio e Ruggi D’Aragona”, 84131 Salerno, Italy; anna.digisi@sangiovannieruggi.it; 6 UOS SPS Direzione Sanitaria, AORN San Giuseppe Moscati, 83100 Avellino, Italy; pierpaolo.disanto@aornmoscati.it

**Keywords:** caregiver burden, cancer caregivers, informal caregivers, measurement instruments, assessment scales, systematic review, nursing

## Abstract

**Background/Objectives**: Caregiver burden is a relevant dimension of the caregiving experience among informal caregivers of cancer patients and is associated with psychological, physical, and social consequences. Although several instruments are available to assess the phenomenon, there is still limited consistency in the tools used across empirical oncology studies. **Methods**: A systematic review was conducted according to the PRISMA 2020 statement. A literature search was carried out in PubMed, Scopus, Web of Science, CINAHL, and PsycINFO up to February 2026. Quantitative studies assessing caregiver burden using standardized instruments in informal caregivers of adult cancer patients were included. **Results**: Thirteen studies met the eligibility criteria. The most frequently used instruments were the Zarit Burden Interview, followed by the Caregiver Burden Inventory. Less frequently used instruments included the Caregiver Reaction Assessment and the Caregiver Burden Scale. Most included studies relied on generic caregiver burden instruments originally developed outside oncology-specific contexts. **Conclusions**: Considerable heterogeneity exists in the instruments used to assess caregiver burden in oncology research. Empirical studies continue to rely predominantly on generic caregiver burden scales, while oncology-specific tools appear to be underused. Greater consistency in instrument selection may improve comparability across studies and support the integration of caregiver assessment into oncology practice.

## 1. Introduction

Caregivers play a crucial role in oncology care, acting as an extension of the healthcare workforce by managing complex clinical, practical, and advocacy tasks. Informal caregiving provided by family and friends represents a fundamental complement to professional care [1,2]. Over time, research has increasingly recognized that cancer affects not only patients but also caregivers, generating a multidimensional burden that includes emotional, physical, social, and financial consequences. In this review, caregiver burden was conceptualized as a multidimensional construct encompassing the emotional, physical, social, practical, and financial strain experienced by individuals who provide unpaid care to patients with cancer [3]. This burden arises from the interaction of socioeconomic factors, individual resources, and care-related stressors, and is strongly associated with reduced psychological well-being and quality of life [4,5]. Different caregiver groups (e.g., partners, children, parents) may experience distinct forms of burden, often linked to daily care demands [6,7].

Empirical evidence shows a high prevalence of caregiver burden, particularly in advanced or incurable cancer, where moderate to severe burden is common and often associated with increased care needs and reduced autonomy of patients. Even in early palliative care, moderate burden and high risk of role strain are observed, supporting the need for systematic assessment and early identification of vulnerable caregivers [7,8,9].

To assess caregiver burden, several instruments have been developed, though with considerable heterogeneity. The Zarit Burden Interview (ZBI-22) is the most widely used tool, while others such as the Caregiver Reaction Assessment (CRA) capture both negative and positive aspects of caregiving [8,10]. Oncology-specific tools, including the CareGiver Oncology Quality of Life questionnaire (CarGOQoL), provide a multidimensional evaluation of caregiver well-being, addressing limitations of earlier instruments [11]. Additionally, growing attention has been given to caregivers’ unmet needs, which are closely linked to distress and quality of life; for example, the Caregiver Needs Screen (CNS) was developed for neuro-oncology settings [12,13].

Despite this variety of tools, caregiver burden remains a complex and inconsistently measured construct, limiting comparability across studies and the development of standardized approaches in oncology [14]. While previous reviews focused on the psychometric properties of caregiver burden instruments, less attention has been given to how these tools are applied in real-world oncology research settings [15]. Therefore, the present review does not aim to re-evaluate the psychometric properties of caregiver burden instruments, but rather to examine how these instruments are used and reported in empirical oncology studies. Understanding which instruments are used in empirical studies, and how measurement-related information is reported, is essential to improve comparability across studies and to inform the integration of caregiver assessment into routine oncology care. Accordingly, this systematic review aims to identify and describe the instruments used to assess caregiver burden in informal caregivers of adult cancer patients and to summarize how these measures are reported and applied in empirical oncology studies.

## 2. Materials and Methods

### 2.1. Study Design

A systematic review of the literature was conducted and reported in accordance with the Preferred Reporting Items for Systematic Reviews and Meta-Analyses (PRISMA) 2020 statement [16]. This systematic review aimed to identify and synthesize the instruments used to assess caregiver burden in empirical studies involving informal caregivers of adult cancer patients, and to summarize the measurement-related information reported in those studies. The review protocol was drawn up in accordance with PRISMA-P and registered a priori on the PROSPERO website (CRD420261320586) [17,18]. During the review process, the focus of the synthesis was refined to emphasize the instruments used in empirical oncology studies and the measurement-related information reported in those studies, without changing the original review objective or eligibility criteria. The research question was the following: “*What instruments have been used to assess caregiver burden in informal caregivers of adult cancer patients, and what instrument-related characteristics are reported in empirical oncology studies*?”.

### 2.2. Eligibility Criteria

The studies were selected based on predefined eligibility criteria, structured according to an adaptation of the PICO/PEO framework, considering population, concept, and context.

#### 2.2.1. Population

Studies involving informal caregivers (family members, partners, friends, or significant others) providing unpaid care to adult patients diagnosed with cancer were included, regardless of cancer type or disease stage. Studies conducted on professional or formal caregivers (e.g., nurses or health care workers) and those involving pediatric populations were excluded when they did not meet the review objectives and eligibility criteria.

#### 2.2.2. Concept and Outcome of Interest

Only studies assessing caregiver burden by standardized quantitative tools or validated scales were included. Therefore, articles reporting the use of standardized questionnaires or burden assessment tools were considered eligible. Studies were considered ineligible if they did not measure the caregiver burden with structured tools, did not report results related to the outcome of interest, or presented only qualitative data without rating scales. Qualitative studies were excluded because the objective of this review was to examine standardized quantitative instruments used to assess caregiver burden, rather than to synthesize subjective experiences of caregiving.

#### 2.2.3. Context

Studies conducted in any cancer setting were included, including hospital, outpatient clinic, home care, and palliative care. Observational quantitative studies, predominantly with a cross-sectional design, were included. The identified tools were grouped in narrative synthesis according to their main characteristics, including domains assessed, scoring modalities, oncological setting, and psychometric properties reported.

### 2.3. Information Sources

A literature search was conducted in the main electronic databases, including MEDLINE via PubMed, Scopus via Elsevier, Web of Science Core Collection via Clarivate, CINAHL Complete via EBSCOhost, and PsycINFO via EBSCOhost. No time restrictions were applied, while only articles published in English or Italian were included. This language restriction was applied for feasibility reasons and may have introduced language bias. The last systematic database search was carried out on 21 February 2026. In order to identify further relevant studies, the bibliographic lists of the included articles were also examined (backward citation searching). In addition, a forward citation search was conducted via Web of Science, applied to the key studies included in the review.

### 2.4. Search Strategy

The search strategy was developed by combining controlled terms (MeSH) and free-text keywords related to informal caregivers, Oncology/Cancer, caregiver burden, and burden assessment tools and standardized instruments. The search strategy was not structured around a clinical intervention but focused on caregiver burden and measurement instruments. An example of the search strategy used in MEDLINE via PubMed was: (caregiver* OR “family caregiver*” OR “informal caregiver*”) AND (cancer OR neoplasm* OR oncology OR tumor*) AND (“caregiver burden” OR burden) AND (scale* OR instrument* OR questionnaire* OR assessment).

The strategy has been adapted for each database, considering the specific syntax and indexed terms available. No published automatic filters or automatic string translation tools were used. The complete search strategies for all databases are provided in Supplementary File S1, publicly available on Zenodo.

### 2.5. Selection Process

All identified records were exported to Zotero bibliographic management software, and duplicates were removed before screening [19]. The selection of studies was conducted by two independent reviewers, who carried out screening evaluations of titles and abstracts, full text of potentially eligible articles, and final selection of the included studies. When discrepancies emerged between the reviewers, they were resolved through discussion and consensus, and a third-party reviewer was involved where necessary. No formal inter-rater agreement statistic was calculated; consistency was ensured through independent screening followed by consensus discussion. Consistency between reviewers was ensured through independent screening and consensus discussion. No automation tools or machine learning classifiers were used in the selection process. The entire identification and selection process was documented via the PRISMA flowchart. Screening was conducted entirely by two independent reviewers.

### 2.6. Data Collection Process

Data extraction was carried out by two independent reviewers using a standardized data extraction form and piloted over a limited number of included studies, in order to improve clarity and completeness. Any discrepancies between the reviewers have been resolved through discussion and consensus; if necessary, a third auditor was involved. When relevant information was missing or unclear (e.g., instrument domains, scoring modalities, or psychometric indications), the authors of the original studies were contacted via email for clarification. No automation tools were used for data extraction, nor software dedicated to extracting information from figures or graphs. It was not necessary to translate articles into languages other than English or Italian.

### 2.7. Data Items

The main outcome for which data were sought was the use of caregiver burden assessment tools in informal caregivers of cancer patients. For each included study, the following data were extracted when available: instrument name, number of items, domains/subscales, scoring approach, oncology setting, and any measurement-related information reported by the authors, such as internal consistency or other psychometric indicators. Data were extracted at timepoints reported in the included studies (e.g., baseline, follow-up, treatment phases, palliative setting), without imposing a minimum follow-up time, given the descriptive and instrumental nature of the review. All results compatible with the outcome domains of interest reported in each study were collected; if there were multiple measurements of the same instrument at different times, this information was recorded separately.

### 2.8. Risk of Bias Assessment

The methodological quality of the included studies was assessed using the Joanna Briggs Institute (JBI) critical appraisal checklist for analytical cross-sectional studies, as all included studies adopted a cross-sectional design. Each study was evaluated across eight methodological domains, including inclusion criteria, study setting description, validity of exposure measurement, standardized outcome measurement, identification of confounding factors, strategies to address confounding, reliability of outcome measurement, and appropriateness of statistical analysis. Disagreements between reviewers were resolved through discussion and consensus.

### 2.9. Effect Measures

Given the descriptive nature of the review, which focused on measurement tools, no quantitative effect measures were defined.

### 2.10. Synthesis Methods

Due to the heterogeneity of the tools and study designs, no meta-analysis was conducted. Results were summarized using a narrative and tabular approach, grouping instruments according to their structural characteristics, domains assessed, oncology setting, and measurement-related information reported in the included studies.

### 2.11. Certainty Assessment

Overall confidence in the evidence was assessed descriptively, considering methodological quality, consistency of results, and robustness of the psychometric properties reported. A formal system such as GRADE was not applied, as the review did not aim at the evaluation of clinical interventions, but at the description and analysis of measurement tools.

## 3. Results

**Figure 1:**
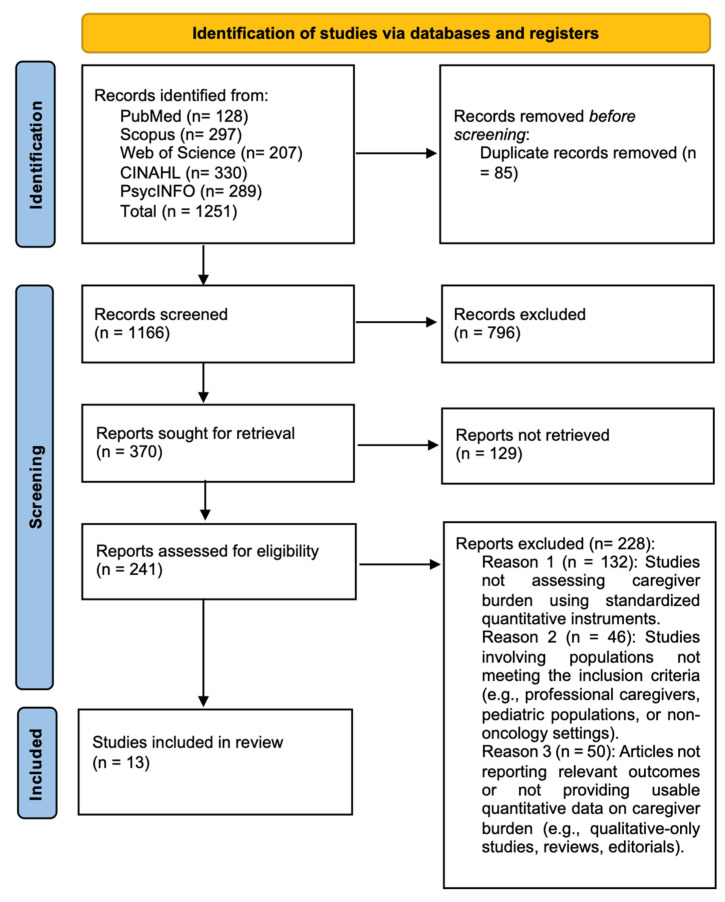
PRISMA flow diagram of study selection process.

The literature search identified 1251 records across the selected electronic databases. After the removal of 85 duplicate records, 1166 records were screened by title and abstract. Of these, 370 reports were sought for retrieval, and 129 reports could not be retrieved. Overall, 241 full-text articles were assessed for eligibility, and 13 studies met the inclusion criteria and were included in the review. The study selection process is presented in the PRISMA 2020 flow diagram (Figure 1).

### 3.1. Characteristics of Included Studies

The studies included were predominantly observational studies with a cross-sectional design, conducted in different geographical and clinical contexts. Sample sizes ranged from 89 to 328 caregivers. The studies were conducted in different care settings, including oncology hospital wards, specialist clinics, palliative care services, and home care. Most of the studies involved family caregivers of patients with different types of cancer and at different stages of the disease, including advanced or palliative stages. The main characteristics of the studies included are summarized in Table 1.

### 3.2. Instruments Used to Assess Caregiver Burden

The included studies used several standardized tools to assess the caregiver burden in caregivers of cancer patients. The characteristics of the main caregiver burden instruments identified in the included studies are summarized in Table 2.

In several observational studies, burden was assessed using the Zarit Burden Interview, one of the most widely used tools to measure the burden of care in caregivers. For example, studies conducted in oncology and palliative care settings have used ZBI to quantify the level of burden and identify associated factors [8,9,10,20,21,22,23,24,25,26,27,28,29]. Similarly, other studies used the Caregiver Burden Inventory to assess the multidimensional aspects of caregiver burden, including physical, emotional, and social components [22,23]. In some specific settings, such as the care of patients with oral cancers, the Caregiver Reaction Assessment (CRA) has been used, which measures different dimensions of the caregiving experience, including disorganization of daily routine and perceived family support [10]. None of the instruments identified in the included studies were specifically developed for oncology caregivers, as most tools were originally designed for broader caregiving contexts.

### 3.3. Instruments Specifically Developed for Oncology Caregivers

Although not identified among the empirical studies included in this review, some instruments have been specifically developed for oncology caregivers in the broader literature. The CareGiver Oncology Quality of Life questionnaire (CarGOQoL) was developed through a rigorous methodological process that included qualitative interviews with cancer caregivers and subsequent psychometric analyses on a large sample of participants [11]. An additional oncology-specific tool is the Caregiver Needs Screen (CNS), designed to identify support needs in caregivers of patients with brain tumors [12].

### 3.4. Measurement-Related Information Reported Across Studies

Limited measurement-related information was reported in the included empirical studies. Most studies clearly described the study population and clinical setting and used validated instruments to assess caregiver burden. Oncology-specific instruments such as CarGOQoL and CNS were identified in the wider literature as examples of tools developed for cancer caregiving contexts, but they were not commonly used in the empirical studies included in this review. Only a minority of studies reported psychometric indicators such as internal consistency (e.g., Cronbach’s alpha) within the study sample, and few studies explicitly justified instrument selection based on measurement properties. This limited reporting reduces the reproducibility of instrument selection and makes it difficult to judge whether the selected tools were appropriate for the specific oncology populations studied.

### 3.5. Synthesized Evidence Across Instruments

Overall, the evidence from the included empirical studies shows that caregiver burden in oncology is predominantly assessed using instruments originally developed for non-oncological caregiving populations. In particular, the Zarit Burden Interview (ZBI), the Caregiver Burden Inventory (CBI), the Caregiver Reaction Assessment (CRA), and the Caregiver Burden Scale (CBS) were the most frequently used tools across the included studies [8,9,10,20,21,22,23,24,25,26,27,28,29]. These instruments were applied in different oncology contexts, including palliative care, advanced cancer, and hospital-based oncology services, confirming their widespread use in caregiver research across different clinical contexts [30,31]. Despite their extensive use, these instruments were not originally developed for cancer caregiving contexts. For example, the ZBI was initially developed to assess burden among caregivers of patients with dementia and has subsequently been applied in a variety of clinical populations [4]. Similarly, the CBI was designed to capture the multidimensional nature of caregiver burden across emotional, physical, and social domains [32], while the CRA evaluates both positive and negative aspects of caregiving, including self-esteem, family support, and financial strain [33]. The CBS, developed in Scandinavian caregiving research, measures general strain, isolation, and emotional involvement among caregivers [29]. In contrast, several instruments have been specifically developed for caregivers of cancer patients. Among these, the CareGiver Oncology Quality of Life questionnaire (CarGOQoL) represents one of the most comprehensive oncology-specific tools, developed through qualitative interviews with cancer caregivers followed by large-scale psychometric validation [11,34]. Another example is the Caregiver Needs Screen (CNS), designed to identify the supportive care needs of caregivers of patients with brain tumors [12]. Although these oncology-specific instruments provide valuable insights into the cancer caregiving experience, they were not commonly identified in the empirical studies included in this review. These findings suggest that empirical oncology research continues to rely predominantly on generic caregiver burden instruments, while oncology-specific tools remain less frequently used despite their availability in the broader literature.

### 3.6. Reporting Biases

**Figure 2:**
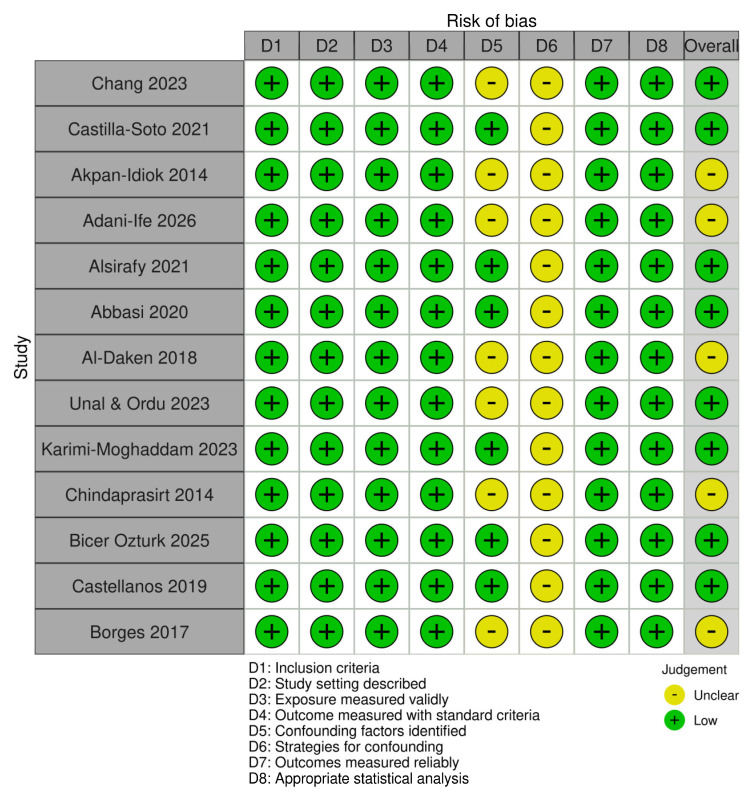
Traffic light plot of methodological quality assessment using the Joanna Briggs Institute (JBI) critical appraisal checklist for analytical cross-sectional studies [8,9,10,20,21,22,23,24,25,26,27,28,29].

All included studies had a cross-sectional design; therefore, the Joanna Briggs Institute (JBI) critical appraisal checklist for analytical cross-sectional studies was applied [35]. Across the eight methodological domains, most studies fulfilled the majority of the appraisal criteria. Specifically, clear inclusion criteria and adequate description of the study setting were reported in all studies. The overall judgement was based on the number and relevance of unmet JBI domains rather than on a numerical cut-off score. Valid and standardized instruments were used to measure caregiver burden in all included studies. However, several studies did not clearly identify potential confounding factors or describe strategies to address them (Figure 2).

Overall, study quality was moderate, with recurrent limitations in confounding control and sampling methods. No studies achieved full compliance across all JBI domains.

## 4. Discussion

This systematic review provides a novel perspective on caregiver burden assessment in oncology by focusing on the real-world application of measurement instruments in empirical studies, rather than their psychometric validation. Our findings show that caregiver burden in oncology is predominantly assessed using generic instruments, particularly the Zarit Burden Interview (ZBI), Caregiver Burden Inventory (CBI), Caregiver Reaction Assessment (CRA), and Caregiver Burden Scale (CBS), while oncology-specific instruments remain less frequently adopted in empirical studies. This approach differs from previous COSMIN-based reviews, as the present study focuses on how instruments are actually applied in oncology research [15]. This finding suggests that, although caregiver burden is widely discussed in oncology, fewer empirical studies explicitly report the use of standardized burden instruments in a way that allows comparison across studies. The predominance of generic instruments raises important methodological considerations. Although these tools allow comparison across different caregiving populations, their suitability in oncology may be limited. The applicability of caregiver burden instruments may also vary across cultural and geographical contexts. Instruments developed in one country or clinical culture may not fully capture culturally specific caregiving roles, family expectations, or support systems in other settings. This may be particularly relevant in international oncology research, where caregiver responsibilities, family structures, and access to formal support services may differ substantially across healthcare systems. Caregiver burden emerges as a central dimension of the cancer caregiving experience and is consistently associated with psychological distress, fatigue, and reduced quality of life, in line with prior literature [5,7,25]. Previous studies have highlighted both the significant impact of caregiving on caregivers’ well-being [7] and the crucial yet often under-recognized role of informal caregivers within oncology care systems [5]. A key finding concerns the distinction between generic and oncology-specific instruments. Although widely used tools such as the ZBI provide valuable information, oncology-specific instruments like the CareGiver Oncology Quality of Life questionnaire (CarGOQoL) may offer a more sensitive and comprehensive assessment of cancer caregiving, capturing unique aspects such as prognostic uncertainty, treatment-related burden, and emotional impact of disease progression [7,11,21,25]. An additional relevant finding concerns the lack of consistency in the conceptual dimension assessed across instruments. While most tools capture general aspects such as emotional strain, physical burden, and social limitations, important oncology-specific dimensions appear to be underrepresented. These include uncertainty related to prognosis, treatment-related complexity, and the evolving nature of caregiving across the cancer trajectory [5,7]. Overall, the variability in measurement tools limits comparability across studies. Moreover, few studies reported detailed measurement-related information, such as internal consistency in the specific sample or justification for instrument selection. This limited reporting reduces confidence in instrument selection and makes it difficult to determine whether the tools used were sufficiently reliable, valid, and appropriate for the specific oncology populations studied. The observed heterogeneity of instruments reflects the multidimensional nature of caregiver burden, which includes psychological, physical, social, and financial components [7,21]. As a result, many studies have adopted tools originally developed for other populations, such as dementia caregivers, adapting them to oncology settings [4]. However, the ZBI remains the most widely used, demonstrating moderate to high levels of burden across different cancer settings, including advanced disease and palliative care [8,19,20]. Similarly, other instruments such as the CBI and CRA have been used to explore multidimensional aspects of caregiver burden [7,10,32,33]. In contrast, oncology-specific instruments such as CarGOQoL and CNS were specifically developed to capture cancer-related caregiving challenges and have demonstrated promising psychometric properties [11,12]. This reflects a gap between methodological development and real-world research practice. Beyond familiarity with generic tools, this limited uptake may be explained by restricted cross-cultural validation, limited availability in multiple languages, lack of integration into routine clinical workflows, and the perceived lower comparability of oncology-specific tools across studies. Additional considerations include the broader comparability of generic instruments across different caregiving populations. Recent literature has also highlighted the need to support caregivers through structured interventions and integrated care pathways [36]. Furthermore, many studies apply validated instruments without re-evaluating their psychometric performance in oncology populations, indicating an additional methodological gap. The methodological limitations identified through the risk of bias assessment also affect the interpretation of findings. In particular, limited control for confounding factors and potential sampling limitations may reduce the generalizability of results and may partly explain variability in caregiver burden estimates across studies. Generic instruments such as the ZBI and CBI offer broad and easily comparable measures of caregiver burden, but they may not fully capture oncology-specific dimensions such as prognostic uncertainty, treatment-related complexity, symptom fluctuations, and changes in caregiving demands across the cancer trajectory. Conversely, oncology-specific instruments may provide greater contextual relevance but appear to be less frequently adopted in empirical studies. Therefore, generic instruments may be appropriate when comparability across populations is prioritized, whereas oncology-specific tools may be preferable when the objective is to capture the distinctive features of cancer caregiving. Instrument selection should therefore be guided by the study aim, clinical context, target population, and domains of burden considered most relevant.

From a clinical perspective, systematic assessment of caregiver burden is essential to identify caregivers at risk of distress and reduced quality of life. Burden is influenced by both patient-related and caregiver-related factors, including disease stage, symptom severity, social support, and duration of care. Integrating caregiver burden assessment into oncology pathways may facilitate early identification of vulnerable caregivers and support the development of targeted interventions [24,36]. Overall, these findings underscore the need for greater standardization in caregiver burden measurement and for increased use of oncology-specific instruments to improve both research comparability and clinical applicability. This gap between instrument development and real-world application represents a key methodological issue in oncology caregiver research. In practice, instrument selection should consider the purpose of assessment, the clinical setting, the caregiver population, the domains covered by the instrument, and the availability of evidence supporting its use in oncology.

### Strengths and Limitations

This review has several strengths. First, it specifically focused on the instruments used in empirical oncology studies involving informal caregivers of adult cancer patients. Second, it provides a structured overview of the main tools actually applied across different oncology settings. Third, it highlights the contrast between the widespread use of generic caregiver burden scales and the limited uptake of oncology-specific instruments. There are also some limitations. First, the included studies were predominantly cross-sectional observational studies, which limits the interpretability of associations reported in the original literature. Second, the heterogeneity of tools, settings, and caregiver populations reduced comparability across studies. Additional limitations should be acknowledged. The restriction to English and Italian studies may have introduced language bias, and potential publication bias cannot be excluded. The relatively small number of included studies reflects both the specificity of the inclusion criteria and the limited reporting of measurement-related information in primary research. Moreover, because this review did not perform a formal COSMIN-based psychometric evaluation, conclusions regarding measurement quality should be interpreted cautiously.

## 5. Conclusions

Caregiver burden is a central dimension of the cancer caregiving experience and is assessed using a variety of instruments in oncology research. This review shows that empirical studies predominantly rely on generic caregiver burden scales, while oncology-specific instruments remain underused. Greater consistency in instrument selection may improve comparability across studies and facilitate the integration of caregiver assessment into routine oncology care. Future studies should prioritize transparent reporting of instrument selection, consider oncology-specific tools when appropriate, and support the development of consensus-based recommendations for caregiver burden assessment in oncology research.

## Figures and Tables

**Table 1:** Characteristics of the included empirical studies.

Study	Country	Study Design	Sample (Caregivers)	Cancer Population	Instrument Used	Key Findings
Chang et al., 2023 [10]	Taiwan	Cross-sectional	107	Oral cancer patients receiving home care	CRA	Burden associated with disrupted schedule and lack of support
Castilla-Soto et al., 2021 [9]	Spain	Cross-sectional	174	Advanced cancer patients in palliative care	ZBI	Moderate caregiver burden during early palliative care
Akpan-Idiok et al., 2014 [20]	Nigeria	Cross-sectional	210	Advanced cancer patients attending oncology clinics	ZBI	High physical, emotional and financial burden
Adani-Ifè et al., 2026 [21]	Togo	Cross-sectional	123	Hospitalized oncology patients	ZBI	Mild–moderate burden linked to disease stage
Alsirafy et al., 2021 [8]	Egypt/Saudi Arabia	Cross-sectional	218	Incurable cancer patients receiving palliative care	ZBI	Significant caregiver burden in advanced cancer
Abbasi et al., 2020 [22]	Iran	Cross-sectional	154	Cancer patients receiving treatment	CBI	Higher burden associated with poorer caregiver QoL
Al-Daken & Ahmad, 2018 [23]	Jordan	Cross-sectional	111	Hospitalized cancer patients	CBI	Burden predicted by sleep disturbance and treatment effects
Ünal & Ordu, 2023 [24]	Turkey	Cross-sectional	328	Stage IV cancer patients receiving chemotherapy	ZBI	Burden linked to emotional distress and coping
Karimi-Moghaddam et al., 2023 [25]	Iran	Cross-sectional	300	Various cancer types in oncology wards	ZBI	Burden associated with anxiety and depression
Chindaprasirt et al., 2014 [26]	Thailand	Cross-sectional	150	Elderly cancer patients with advanced disease	ZBI	Moderate caregiver burden reported
Bicer Ozturk et al., 2025 [27]	Turkey	Cross-sectional	162	Patients undergoing oncological surgery	ZBI	Burden associated with health anxiety and depression
Castellanos et al., 2018 [28]	USA	Observational	89	Head and neck cancer patients	ZBI	Caregiving task burden associated with psychological distress
Borges et al., 2017 [29]	Brazil	Cross-sectional	91 dyads	Lung cancer patients	CBS	Burden associated with patient quality of life

**Table 2:** Caregiver burden instruments identified in the review.

Instrument	Oncology-Specific	Number of Studies	Main Domains
Zarit Burden Interview (ZBI)	No	8	Impact of caregiving, interpersonal strain, emotional burden, role strain
Caregiver Burden Inventory (CBI)	No	2	Time-dependence burden, developmental burden, physical burden, social burden, emotional burden
Caregiver Reaction Assessment (CRA)	No	1	Caregiver self-esteem, disrupted schedule, financial problems, lack of family support, health problems
Caregiver Burden Scale (CBS)	No	1	General strain, isolation, disappointment, emotional involvement, environmental burden

## Data Availability

All data supporting the findings of this study are included within the manuscript and its Supplementary Materials. Additional information is available from the corresponding author upon reasonable request.

## Supplementary Materials

Supplementary File S1. Full Search Strategies available on Zenodo (https://doi.org/10.5281/zenodo.20095013, accessed on 19 March 2026).

## Public Involvement Statement

No public involvement in any aspect of this research.

## Guidelines and Standards Statement

This manuscript was drafted against the Preferred Reporting Items for Systematic Reviews and Meta-Analyses (PRISMA) 2020 statement for systematic reviews [16].
